# Bioluminescence imaging for IL-1β expression in experimental colitis

**DOI:** 10.1186/1476-9255-10-16

**Published:** 2013-04-11

**Authors:** Limei Li, Zhenzhe Liu, Xinyu Yang, Huimin Yan, Shisan Bao, Jian Fei

**Affiliations:** 1 School of Life Science and Technology, Tongji University, Shanghai 200092, PR China; 2 Shanghai Research Centre for Model Organisms, Shanghai 201203, PR China; 3 Discipline of Pathology and Bosch Institute, School of Medical Sciences, University of Sydney, Sydney, NSW, 2006, Australia; 4 State Key Laboratory of Cell Biology, Institute of Biochemistry and Cell biology, Shanghai Institutes for Biological Sciences, Chinese Academy of Sciences, Shanghai, 200031, PR China; 5 Currently working at: State Key Laboratory of Cell Biology, Institute of Biochemistry and Cell biology, Shanghai Institutes for Biological Sciences, Chinese Academy of Sciences, Shanghai, 200031, PR China

## Abstract

**Background:**

Interleukin 1 beta (IL-1β) contributes to the development of inflammatory bowel disease (IBD) and is correlated with the severity of intestinal inflammation. However, the precise source of IL-1β producing cells in DSS colitis is currently not known.

**Methods:**

To determine IL-1β activity during intestinal inflammation in real time, an IL-1β transgenic mouse has been generated by incorporating the firefly luciferase gene driven by a 4.5-kb fragment of human IL-1β gene promoter (named cHS4I-hIL-1βP-Luc transgenic mice). Dextran sodium sulfate (DSS) induced colitis was confirmed with clinical presentation and histopathology.

**Results:**

A substantial increase in luciferase activity (reflecting IL-1β production) in the region of inflamed colon was observed in a time dependent manner, followed by additional activity in the region of the mesenteric lymph node. The up-regulated luciferase activity was suppressed by dexamethasone (steroids) during DSS challenge, consistent with reduced severity of colitis, confirming the specificity of luciferase activity.

**Conclusions:**

Our data suggests that bioluminescence is an interesting technology, which may be used to evaluate transcription of various genes in real time in experimental colitis.

## Background

Inflammatory bowel disease (IBD), encompassing Crohn’s disease (CD) and ulcerative colitis (UC), is recognized as a widespread, debilitating condition with increasing incidence in Western societies in both children and adults [1-3]. The natural history of IBD is characterized by relapse and remission, with several factors known to trigger relapses including infection, ingestion of non-steroidal anti-inflammatory drugs and changes in smoking habits [4]. The aetiology of IBD is still not fully understood, despite decades of extensive research. It is believed that the imbalance of pro-inflammatory and anti-inflammatory cytokines contributes to the development of colitis [5-7].

Interleukin-1β, primarily secreted by monocytes and macrophages upon activation is one of the main drivers of inflammation. Macrophages are recruited and activated from peripheral blood into the inflamed colon [8,9]. IL-1β stimulates the production of inflammatory eicosanoids that subsequently induce neutrophil - chemoattractant and neutrophil-stimulating [10]. Released mature IL-1β protein resulting from inflammatory stimulus at the injured tissue, together with other cytokines and mediators (e.g. oxygen radicals) cause a cascade of inflammatory responses and tissue damage [11,12]. The binding between IL-1 and IL-1 receptor activates the NF-κB signal-transduction pathway [13], resulting in the up-regulation of other pro-inflammatory mediators such as TNF, IL-6 and IL-12 [14]. IL-1β is one of the key mediators of intestinal inflammation in IBD with a role in amplifying mucosal inflammation [15,16], consistent with the finding that IL-1β is up-regulated in IBD patients [17] and animal models [18,19]. On the other hand, IL-1β receptor antagonist reduces inflammation in an animal model of colitis [18,19].

IL-1β in inflamed intestine is mainly produced by infiltrating lamina properia monocytes including macrophages in the IBD mucosa [16]. However, IL-1β can also be produced by intestinal smooth muscle cells and fibroblasts [20]. The precise source of IL-1β producing cells in our animal model will be investigated in our future experiment.

Animal models of experimental colitis have been useful in confirmation of these clinical observations [11,21,22]. Furthermore, developing a method to monitor real time IL-1β activity *in vivo* would provide a unique opportunity to assess the precise progression of intestinal inflammation, using a DSS induced colitis model.

In this paper, colitis was induced using dextran sodium sulfate (DSS) in a cHS4I-hIL-1βP-Luc transgenic mouse, in which the expression of luciferase reporter gene was under the control of the human IL-1β gene promoter [23,24]. A “biophotonic” imaging system equipped with a highly light-sensitive camera allows non-invasive study of the transcriptional activity of IL-1β gene promoter in real time during the development of IBD, which could be used to evaluate the effects of anti-inflammatory compounds on IL-1β gene induction *in vivo*.

## Methods

### Genotyping of cHS4I-hIL-1βP-Luc transgene in mice

cHS4I-hIL-1βP-Luc transgenic mice, generated in the C57/B6 × CBA background [23,24], were backcrossed to C57/B6 for 3 generations before the experiment. Transgenic founders and their offsprings were identified by PCR using the forward-luc (5^′^ TTCCGCCCTTCTTGGCCTTTATGA 3^′^) and reverse-luc (5^′^ CAGCTATTCTGATTACACCCGAGG 3^′^) primers specific for the luciferase gene. All animals were housed under conventional laboratory conditions with food and water *ad libitum.* Experiments adhered to the guidelines of the local institutional animal care and use committee.

### Induction of colitis

Adult (10 week old, male) cHS4I-hIL-1βP-Luc transgenic mice were given *ad libitum* 3% w/v dextran sulphate sodium (DSS, MW 36 000–44 000; MP Biomedicals, CA, USA) dissolved in tap water for four consecutive days, as described [11,12,22], while control groups received tap water only. On day five, the DSS solution was replaced with water, allowing some degree of colonic epithelial recovery. To confirm that the luciferin activity was inflammation specific, the mice were challenged with 3% DSS in drinking water and also dexamethasone (St. Louis, MO, USA,1.5 mg/mg) i.p. daily for five days. The luciferase signal was imaged and compared with that of the control group, which was injected with saline.

### Assessment of the extent of experimental colitis

To confirm the severity of the colitis model, DSS-induced colitis was evaluated by body-weight and stool score daily. Weight loss on each day was calculated as the percentage of the baseline of bodyweight. Blood loss and stool consistency were scored, based on our previous description [11,12,22]. Scores were defined as follows: Blood loss: 0 = negative, 2 = positive, 4 = gross bleeding. Stool consistency: 0 = normal, 2 = loose stools, 4 = diarrhea.

### Histopathologic analysis

For histopathologic analysis at day 6 (2 days after the end of DSS challenge), transverse colon was collected and fixed in 10% buffered formalin phosphate, embedded in sucrose, frozen in dry ice using optimal cutting temperature (OCT) compound and cryosectioned. Cross sections were stained with hematoxylin/eosin (H&E, Lerner, New Haven, CT). Histopathological scores were used to quantify the intestinal inflammation, as described previously [11,12,22].

### *In vivo* and *ex vivo* imaging

*In vivo* bioluminescent imaging was performed using an IVIS imaging system (Bio-Real, QuickView3000, Austria). At the selected time points, the mice were anesthetized with isoflurane/oxygen, then were injected i.p. with substrate luciferin (Biosyth, Basel, Switzerland) dissolved in PBS (15 mg/ml) at a dose of 150 mg/kg. After 12 min of luciferin injection, images were taken on the imaging stage for 1–5 min. Photons emitted from specific regions were quantified using a LivingImage software (Bio-Real, QuickView3000, Austria). *In vivo* luciferase activity was presented in photons emitted per second.

For *ex vivo* imaging, the experimental mice were scarified at the days 4, 6 and 8 post DSS challenge. The colon, MLN and spleen were collected and were imaged with the same settings used for the *in vivo* studies on a heated stage in the IVIS system.

### Statistics

All data are expressed as means ± SE. The data were analysed by one-way ANOVA. A P value of < 0.05 was considered significant.

## Results

### DSS induced acute colitis

**Figure 1:**
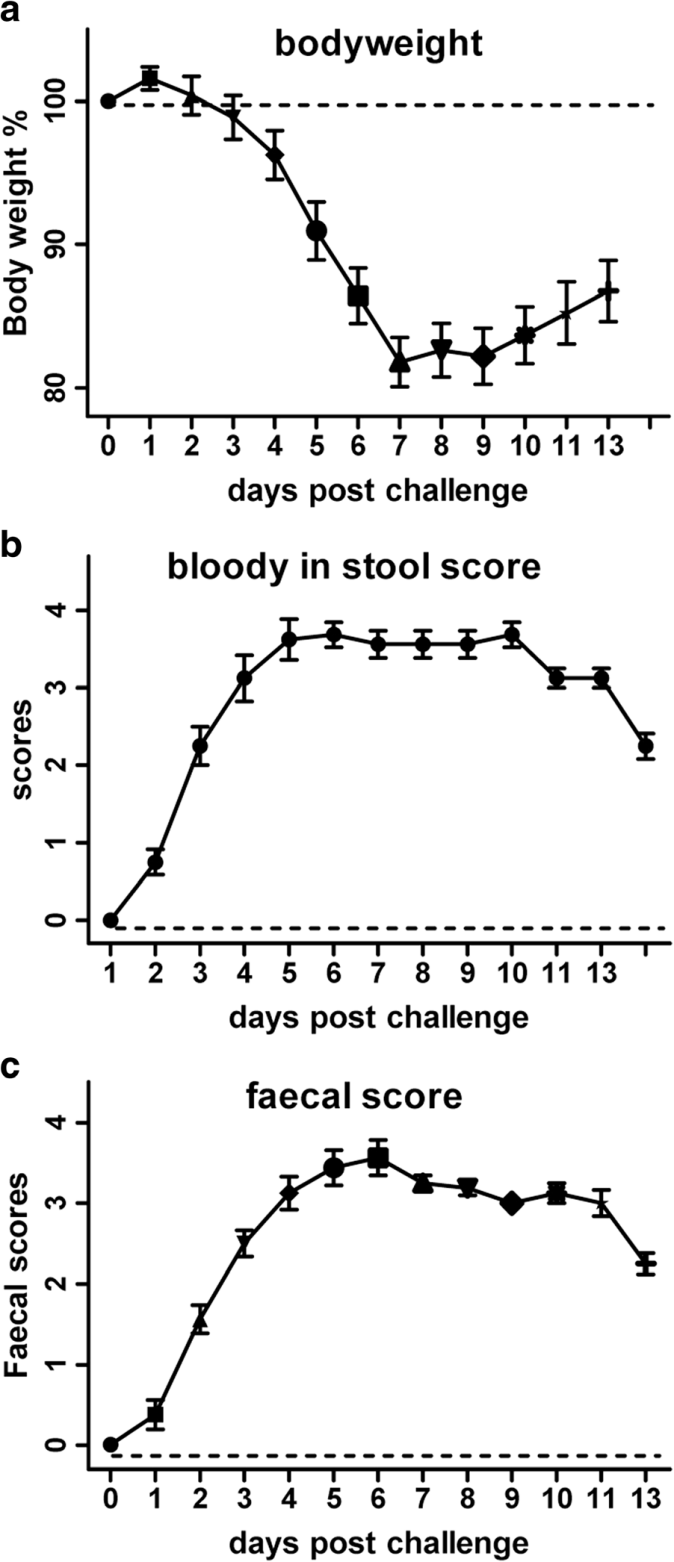
**Clinical and faecal scores. **Body weight changes from 100% baseline (**a**), blood appearance in faeces (**b**) and faecal score (**c**) over 5 d of DSS oral challenge commenced at the period of 13 days in cHS4I-hIL-1βP-Luc transgenic mice. Mean ± SEM. * P<0.05, ** P< 0.01, *** P< 0.001.

Mice body weight dropped gradually from day 4, to a low at day 7 (17.5%) (p<0.001) post DSS challenge, before slowly gaining again 3 days after cessation of DSS (Figure 1a). The body weight loss was consistent with faecal blood scores and faecal scores, showing progressive diarrhoea and bloody stools starting from day 2 and peaking at day 5 post DSS challenge (Figure 1b and c). These scores plateaued from day 5 till 10 and gradually dropped on day 13. The large intestine was collected immediately on day 6 following DSS. Macroscopic gross bleeding was observed in some areas of the colon from all DSS treated animals. Furthermore, colon weight was increased by about 30%, but colon length was reduced about by 30%, compared to water treated controls (data not shown). Histological examination of the distal colon of DSS treated mice showed transmural inflammation involving all layers of the bowel wall, with a marked increase in the thickness of the muscular layer, adherence to surrounding tissues, mucosal ulceration, pronounced depletion of goblet cells, reduction of the density of the tubular glands, disseminate fibrosis, and focal loss of crypts (data not shown) with substantial leucocyte infiltration. In contrast, no abnormality was observed in the colon of mice without DSS challenge (data not shown).

### Luciferase expression induced by DSS in cHS4I-hIL-1βP-Luc transgenic mice

**Figure 2:**
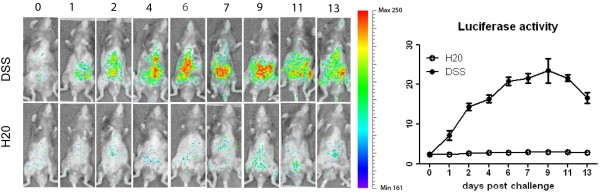
**Luciferase activity in the animals ± DSS challenge *in vivo*. **Luciferase activity in the animals ± DSS challenge was quantified using an IVIS imaging system. The distribution and intensity were recorded at the different day following DSS or H_2_0 challenge.

Constitutive levels of IL-1β production (luciferase expression) in the abdominal region of cHS4I-hIL-1βP-Luc transgenic mice were determined at day 0. IL-1β (luciferase expression) production was up-regulated gradually following DSS challenge, starting on day 1 and peaking on day 9. The magnitude of the increase was 3.8, 6.9, 7.3, 9.8, 10.3 and 11.1-fold on days 1, 2, 4, 6, 7, and 9 days post challenge, respectively (Figure 2). IL-1β (luciferase expression) was observed throughout the abdominal region prior to challenge. Following DSS challenge, the distribution was denser and localised near the centre/left lower quadrant of the abdomen on day 1. The density was up-regulated gradually from day 2 and appeared close to plateau by day 6, with the distribution seemingly localised to the proximal colon. Condensed but strong distribution was observed on days 7 to 9, but then slowly decreased on days 11 to 13, accompanied by more diffuse distribution (Figure 2). The kinetics and magnitude of IL-1β signals (luciferase expression) were consistent with previously published expression patterns of endogenous IL-1β mRNA [25].

**Figure 3:**
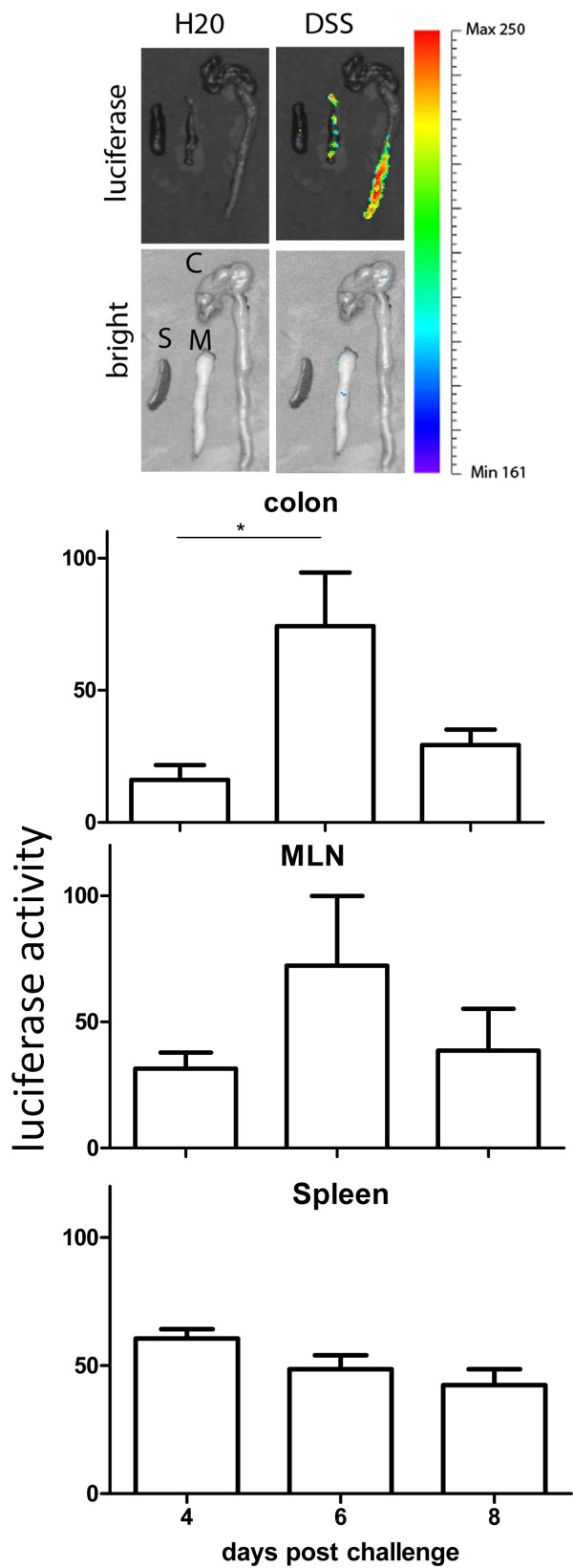
**Luciferase activity in the colon, MLN and spleen following DSS challenge *ex vivo.*** The level of luciferase activity driven by the promoter of IL-1β was quantified. Luciferase activity was confirmed *ex vivo *in the colon, MLN and spleen following DSS challenge at the days 4, 6 and 8 with quantification. Mean ± SEM. * P<0.05, ** P< 0.01, *** P< 0.001.

To confirm the luciferase activity detected *in vivo* was mainly from the inflamed colon, as well as clarify the kinetics of IL-1β, ex vivo luciferase activity from the colon, MLN and spleen were evaluated by live imaging on days 4, 6 and 8 post DSS challenge. IL-1β expression was ~2-, 8- and ~3-fold greater in the colon from DSS challenged animals compared to that from mock challenged (Figure 3). This difference was significant at days 4 and 6 (P<0.05), but not at day 8. A similar pattern of IL-1β expression was observed in the MLN from DSS challenged individuals, but no significant difference was detected among the days 4, 6 and 8. No significant difference in IL-1β expression was detected in the spleen with DSS challenge at various time-points, despite showing the highest level on day 4 post challenge (Figure 3).

**Figure 4:**
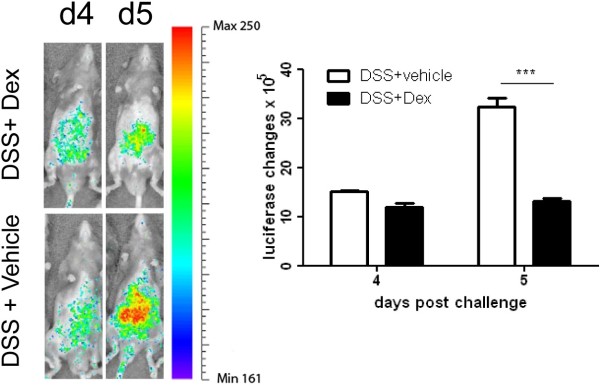
**Luciferase activity in the animals with DSS challenge ± Dex treatment *in vivo. ***Luciferase activity in the animals with DSS challenge ± Dex treatment was quantified using an IVIS imaging system. The distribution and intensity were recorded at the days 4 and 5 following DSS challenge. Luciferase activity was suppressed in the mice with Dex treatment. Mean ± SEM. * P<0.05, ** P< 0.01, *** P< 0.001.

Dexamethasone, a synthetic glucocorticoid, has been well characterized for its ability to inhibit inflammation. To confirm that up-regulated luciferase activity is IL-1β specific, dexamethasone was used alone and as a co-treatment with DSS (1.5 mg/kg). There was a 20% or ~60% reduction in luciferase activity in the dexamethasone and DSS co-treated mice as compared with DSS treated mice on day 4, or day 5 (Figure 4).

## Discussion

Our DSS-induced model of colonic inflammation displays pathological and clinical similarities to human colitis and has been widely used for pharmacological and pathophysiological studies [11,21,25,26]. DSS induced colitis results from the influx of bacteria into the lamina propria, due to an alteration of the colonic inner mucus layer [11]. The inflammatory cytokine, IL-1β, plays a major role in intestinal inflammation development and increased dramatically in the colonic mucosa during disease [27,28]. Our results are in agreement with these findings, showing an increase in luciferase expression driven by IL-1β gene promoter in the inflamed colon. The luciferase activity was significantly higher in the intestine, and relatively higher expression levels were also seen at the position of the mesenteric lymph node. These *ex vivo* data were consistent with previous studies.

Conventional methods for monitoring IL-1β gene expression rely on either measuring circulating levels of IL-1β in the serum or mRNA expression in tissues. Compared with these methods, the approach reported in this study is convenient and sensitive, while allowing less animals to be used. Moreover, this approach offers kinetic quantification and information on the anatomical distribution of IL-1β gene expression.

IL-1β, scarcely distributed throughout the abdominal region prior to challenge, was up-regulated and broadly distributed, but most densely observed near the centre/left lower quadrant of the abdomen on day 1, which supports that DSS gradually induced intestinal inflammation. The increased IL-1β appeared close to plateau by day 6 with the distribution seemingly shifted to the proximal colon, which is line with induced colitis present after 5 days of DSS challenge. Condensed but strong distribution was observed on days 7 to 9, but then slowly decreased on days 11 to 13, accompanied by more diffuse distribution (Figure 2). Our *ex-vivo* data showed that IL-1β was highest in the colon at day 6, but declined at day 8 (Figure 3), supporting that the main source of induced IL-1β is in the inflamed colon *in vivo.* A similar pattern of IL-1β in MLN *ex vivo* suggests IL-1β producing leucocytes migrated into the draining lymph nodes. No significant increase present in the spleen suggests that DSS induced acute colitis focuses on the gut rather than systemic inflammation.

Despite DSS challenge ceasing on day 5, IL-1β activity continued to increase, peaking at day 9 following DSS challenge, consistent with faecal and blood scores over this time frame. This data suggests that IL-1β activity correlates with the severity of colitis, confirmed with histopathological findings, making the bioluminescence model a reliable method for monitoring inflammation.

Despite the robustness of our bioluminescence model, we acknowledge that there remain a number of limitations. Currently, this model has limited resolution and cannot pinpoint the exact source of IL-1β at the cellular level. Furthermore, it doesn’t provide information about IL-1β translation and whether this correlates with observed transcriptional changes. In future experiments tissues will be collected at different time points for detection of IL-1β protein, using Western blot and immunohistochemistry to confirm the relationship between transcription and translation.

The observed dexamethasone dependant reduction of IL-1β expression suggests that this model can be used to evaluate the efficacy of medical therapy by showing decreased expression of inflammatory mediators. The bioluminescence/IL-1β model could be also used to study experimental therapies in IBD [29] such as faecal transplantation [30], which is an alternative therapy in treatment of IBD with promising outcomes.

## Conclusions

Compared with the traditional method to monitor IL-1β gene expression in DSS induced experimental colitis, bioluminescence is an interesting technology that may be used to evaluate transcription of various genes in real time in experimental colitis.

## Competing interests

All the authors declare that there is non-financial competing interest.

## Authors’ contributions

LL and ZL performed the major part of the experiment. XY and HY performed partial live imaging experiments. SB contributed intellectual input to the experiments and wrote manuscript. JF designed the experiments and coordinated the project. All authors read and approved the final manuscript.
